# Pain assessment using physiological responses/markers in different types of pain: a scoping review

**DOI:** 10.1038/s41746-025-02241-6

**Published:** 2026-01-15

**Authors:** Camila Camacho-Navas, Ling Li, Kavita Poply, Vivek Mehta, Panicos Kyriacou

**Affiliations:** 1 Research Centre for Biomedical Engineering, City St George’s, University of London, London, UK; 2 Pain Research Centre, Barts Health NHS Trust, London, UK; 3 Queen Mary University of London, London, UK

**Keywords:** Neurophysiology, Diagnostic markers, Biomedical engineering, Autonomic nervous system

## Abstract

Pain is a complex multidimensional experience that integrates sensory and emotional components, presenting significant challenges for accurate assessment in clinical practice. Traditional methods of pain evaluation rely on subjective self-reporting and each individual’s ability to communicate their pain experience. In light of the effect of pain on the Autonomic Nervous System, researchers are interested in developing objective assessment techniques using physiological signals. This paper outlines the latest advances in pain biomarkers and machine learning methods for assessing pain using physiological signals, highlighting the growing interest and unmet demand in this area. A comprehensive literature review was conducted, covering studies between 2014 and 2024. The discussion is organised into two areas: first, an analysis of the variations in signal feature behaviour across different pain types, and second, a review of the current state-of-the-art models for pain assessment developed using classical machine learning and deep learning techniques.

## Introduction

The International Association for the Study of Pain (IASP) defines pain as “an unpleasant sensory and emotional experience associated with, or resembling that associated with actual or potential tissue damage”^1^. Pain perception involves complex interactions between the nervous system, the brain, and emotional factors, making it both a physical and psychological phenomenon. The sensory component encompasses nociception, the neural pathways transmitting pain sensation, and the physiological encoding and processing of those signals. The emotional component involves the individual’s anticipation of potential harm and the unique perception of pain^1–3^.

Due to this complexity, objective pain evaluation remains one of the biggest challenges that healthcare providers face in achieving optimal pain management^4,5^. The current standard of clinical practice is the use of self-assessment unidimensional tools like Visual Analog Scales (VAS), Numerical Rating Scales (NRS), and Verbal Rating Scales (VRS)^2^. Additionally, multidimensional questionnaires are often employed to capture broader aspects of the pain experience, including its quality, emotional impact, and interference with daily activities^5^.

Despite being simple and easy to use to gather insightful information from the patient, these methods have some limitations. They rely on the user’s ability to understand and communicate their pain perception, and the response provided may be influenced not only by the sensory information but by different social and psychological factors like anxiety, pain catastrophising and sensitivity^3,6^.

Objective pain assessment using physiological signals is an increasingly promising field. It is grounded in the premise that pain triggers changes in the Autonomic Nervous System (ANS). The ANS plays a fundamental role in regulating basal autonomic functions and biological processes such as blood flow, digestion, and respiration, leading to changes that can be measured using physiological signals^7–10^.

Despite extensive efforts to develop an objective tool for assessing pain, no objective method for pain assessment has been widely accepted or integrated into standard clinical practice. Previous reviews have explored these efforts, often focusing on specific pain types or technologies individually. For instance^7^, focused on the identification of relevant sensor technologies and analytical techniques specifically for acute pain, while^11^ discussed key signatures for chronic pain assessment, concentrating solely on Functional Magnetic Resonance Imaging (fMRI) and electroencephalography (EEG). Taking a technology-specific approach^12–14^, [13] aimed for the identification of photoplethysmography (PPG) derived indexes exclusively for the assessment of postoperative pain, and [14] provided an overview of signal processing methods and machine learning (ML) techniques used with electrodermal activity (EDA) in pain assessment.

However, to the best of our knowledge, no comprehensive review has examined how physiological signals and ML techniques are applied across different pain types, nor has one compared the behavioural and physiological signatures that distinguish these conditions. This review aims to address that by analysing the use of physiological signals and ML models in pain assessment, with a particular emphasis on identifying patterns and differences in behavioural features across acute, chronic, and perioperative/postoperative pain contexts.

## Results

### Search results

**Fig. 1:**
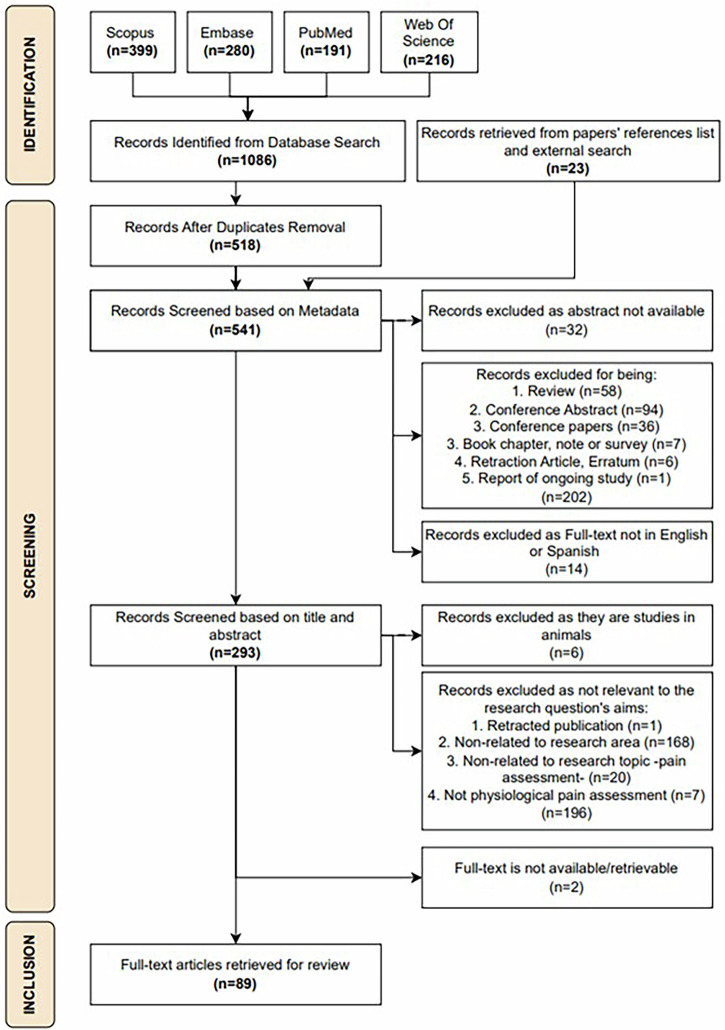
Flow diagram illustrating the search strategy based on PRISMA-ScR guidelines. The figure presents the flow of information through the different stages of the systematic review. It summarizes the number of records identified by the search strategy, the screening steps applied, and the reasons for exclusion, leading to the final set of studies included.

Figure 1 outlines the identification and selection process for the research papers retrieved. After duplicate removal, the search strategy yielded 518 scientific publications from the databases searched. Additionally, by screening the reference list of all literature reviews and relevant publications found, 23 papers were manually included, for a total of 541 papers screened against the inclusion criteria. Finally, after screening the metadata and abstracts, 89 papers were identified as meeting the criteria for inclusion in the review.

**Fig. 2:**
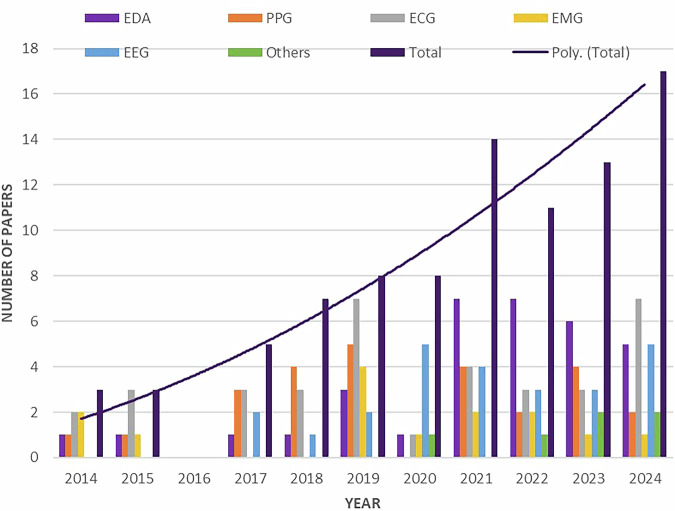
Trends in the Use of Physiological Signals for Pain Assessment Research from 2014 to 2024. The figure illustrates the publication trends on the use of physiological signals in pain assessment over the past decade. Individual categories represent specific physiological signals, while the “Others” category includes all signals reported in the literature that were not assigned to a predefined group. The polynomial line labelled “Poly. (Total)” depicts the overall trajectory of publications addressing pain assessment with physiological signals during this period.

A preliminary analysis of publication trends through the years reveals a growing interest among researchers in assessing pain through physiological signals. This increased interest is illustrated in Fig. 2, which shows that the number of publications retrieved went from 3 in 2014 to 17 in 2024.

In total, the included studies reported a wide variety of physiological signals for pain assessment. The most investigated signals were electrocardiography (ECG) (*n* = 36 studies), where HRV features were the most widely discussed, followed by EDA (*n* = 33 studies), PPG (*n* = 26 studies), and EEG (*n* = 25 studies). Less frequently reported were electromyography (EMG) (*n* = 14 studies) and other signals, such as blood pressure (BP) in four studies, respiration rate (Resp) in four studies, Functional Near-Infrared Spectroscopy (fNIRS) in three studies, and pupillometry in two studies, which implemented those signals. The lower frequency of reports containing EMG and other signals could have been due to the nature of the search strategy, where the keywords did not include those signals strictly. Notably, several studies simultaneously recorded and analysed multiple physiological signals, either in combination or separately. Therefore, the reported frequencies do not sum to 100% of the included studies but rather reflect the number of times each signal appeared across the literature.

Among the 89 studies included, 36 papers reported the use of machine learning approaches for pain assessment. The remaining 53 papers focused solely on traditional statistical methods to validate and analyse the behaviour of various physiological signals and specific markers related to pain assessment.

### Currently available commercial devices

From the reviewed studies, two commercial devices were identified as currently used for pain monitoring: the Medasense PMD-200 system^15,16^, and the Newborn Infant Parasympathetic Evaluation (NIPE) Monitor^17^.

The PMD-200 system is a physiological monitor developed by Medasense to assist clinicians with analgesic dosage for patients under general anaesthesia. It provides a numeric scale of nociceptive response levels, known as the Nociception Level (NOL) Index. This numeric non-linear value is generated by a machine learning model trained using the random forest algorithm, which takes PPG, EDA, peripheral temperature and accelerometry as the input to calculate the NOL Index^18^.

Recent studies have analysed the usefulness of this technology for pain assessment in awake individuals, where other factors besides nociception might activate autonomic nervous system responses. The NOL Index has shown a significant, mild positive correlation with nociception and the potential to discriminate between nociceptive vs non-nociceptive stimulation in postoperative patients^15^. It has also shown a mild correlation with acute pain in chronic pain patients^16^. However, this might not be suitable for assessing moderate acute pain levels or discriminating chronic pain^15,16^.

Similarly, a NIPE Monitor has been commercialised by MDoloris Medical Systems. This technology is specifically designed to evaluate intraoperative and postoperative pain in infants. It analyses continuous cardiac signals derived from ECG or PPG waveform and provides an index between 0 and 100^19^. This monitor was recently used to perform a comparison and characterisation of intraoperative and postoperative pain after open vs laparoscopic inguinal hernia repair in children under two years^17^.

### Open access physiological databases for pain study

Based on the outcomes of the reviewed studies, summarised in Table 1, three publicly accessible databases containing physiological recordings for pain analysis were identified. The BioVid Heat Pain database was the most frequently used, appearing in nine of the included papers. The X-ITE Pain database was reported in three studies, while the PainMonit database was used in one.

**Table 1:** Studies for Pain Assessment Using Physiological Signals

Signals	Type of Pain	Subjects	Sample Size M=Male F=Female	Age (Mean ± SD or range)	Pain Modulation Method	Physiological Markers Studied	Self-Report	Author, Year
**EMG**	Acute	Volunteers	40 (20M 20F)	Not provided	(S) Electrical: electrodes. Thermal -heat-: water	NFR	NRS	Jurth et al., 2014^26^
**ECG**	Acute	Patients	Not provided	Not provided	Contractions during labour	ECG frequency spectrum analysis	VAS	Chu et al., 2024^91^
		Volunteers	52 (26M 26F)	18-36	(S) Thermal -heat-: thermode block	HRV (RRI, LnRMSSD, LF, HF)	N/A	Tracy et al., 2018^40^
		Volunteers	123 (48M 75F)	22.43 ± 2.94	(S) Thermal -cold-: CPT	HRV: Log-transformed LF and HF	NRS	Umeda & Okifuji^39^
	Chronic: chronic musculoskeletal, neuropathic, …	Patients	363 (119M 244F)	56.3 ± 14.01	Chronic pain resting state	HRV: IBI, HR, SDNN, RMSSD, pNN50, HRV triangular index, TINN, LF, HF, LF/HF	VAS / NRS	Moens et al., 2023^37^
	Perioperative	Patients	80	Not provided	Surgery	ANI	EAPS	Jean et al., 2022^97^
	Post-operative	Patients	107 (46M 61F)	37.4 ± 15	Postoperative pain	ANI	NRS	Abdullayev et al., 2019^35^
	Post-operative: caesarean	Patients	40 (40F)	Not provided	(T) Pharmacologic: analgesics	HR, sympathetic tone, vagal tone	VAS	Deepika & Brindha, 2015^106^
**PPG**	Acute	Volunteers	22 (10M 12F)	27 ± 4.19	(S) Electrical: TENS	Time and freq domain feat., PRV (HF, LF, VLF, NN50, pNN50, NN20, pNN20, SDSD, RMSSD, SDNN)	VAS	Khan et al., 2023^93^
	Chronic: radiculopathy, NSBP, FBSS, CRPS	Patients	20 (15M 5F)	52.25 ± 9.77	(T) Neuromodulation: SCS	PPGA number of changes, PRV (PR min, PR max, PR median, SDNNI, SDANN, RMSSD)	NRS	Patterson et al., 2023^88^
	Nociception / Post-operative	Patients	242 (117M 125F)	58.4 ± 10.6	Pre vs postsurgical pain	60 (median, max and min values) morphological, spatiotemporal, interval and dynamic feat.	NRS	Ryu et al., 2024^54^
	Post-operative	Patients	65 (28M 37F)	50.5 ± 10.5	Pre vs postsurgical pain	PA, Asys, Adia, TriA, TriAsys, and TriAdia, ACAdia, ACAbl, Lsys, Ldia, RS, LRS, RSmax, FS, LFS, Pulse Width, PPIsys, PPIdia, ACVsys	VAS	Yang et al., 2018^47^
		Patients	89 (41M 48F)	54.4 ± 12.9	(T) Pharmacologic: oxycodone	PPGA, PAnorm, SYSvar, DIAvar, HBIvar, PA	NRS	Cho et al., 2018^53^
		Patients	100	53 ± 12.4	Pre vs postsurgical pain	Pulse height, rise time, fall time, average HR, PRV (HF, LF, VLF, LF/HF, AVNN, SDNN, RMSSD, NN20, pNN20, NN50, pNN50)	NRS	Lim, 2019^56^
		Patients	100 (44M 56F)	53.4 ± 12.5	(T) Pharmacologic: oxycodone	2D spectrogram. SPI	VAS	Choi et al., 2021^107^
		Patients	99 (99F)	Not provided	(T) Pharmacologic: tramadol	SPI	NRS	Hamed et al., 2024^108^
**EDA**	Acute	Patients	20 (11M 9F)	54.45 ± 17.44	(S) Electrical: TENS. Mechanical: walk, cough, sit up	SCR (number of peaks, mean, max, range, dPhEDA, IQR, RMS, mean minima, mean maxima) using cvxEDA	NRS	Aqajari et al., 2021^64^
		Volunteers	40 (23M 17F)	25	(S) Electrical: TENS	Amplitude of SCR, SPR, and SSR: SPRET; SCR_Trise	NRS	Bari et al., 2018^60^
		Volunteers	23 (11M 12F)	24.5 ± 4.8	(S) Thermal -heat/cold-: grill	SCR, SCL, and dPhEDA from cvxEDA and sparsEDA approaches. TVSymp and MTVSymp	VAS	Posada-Quintero et al., 2021^61^
		Volunteers	16 (7M 9F)	25.6 ± 4.8	(S) Electrical	SCR, SCL, and dPhEDA from cvxEDA, sparsEDA, CDA, DDA, and DCM approaches. TVSymp and MTVSymp.	NRS	Kong et al., 2021^63^
		Volunteers	23 (23M)	Not provided	(S) Thermal -cold- and intradermal capsaicin(T) Analgesics, placebo	SCL	VAS	Bhatkar et al., 2022^62^
		Volunteers	29	Not provided	(S) Thermal -cold-: CPT	SCL (mean, SD, range, AUC) and SCR (mean, SD, range, peak max, peak min, peak sum, peak num, duration mean, slope mean, AUC) from cvxEDA. Whole signals	NRS	Pouromran et al., 2022^65^
		Volunteers	38 (17M 21F)	Not provided	(S) Thermal -heat/cold-: grill	SCR, TFS-phEDA from cvxEDA	N/A	Pinzon-Arenas et al., 2023^66^
		Patients	53 (26M 27F)	36.2	(S) Thermal -cold-. Electrical (dental)(T) Local anaesthesia	TVSymp (max, peak, mean)	VAS	Tran et al., 2023^69^
	Acute / Chronic: TMJ disorders	Volunteers / Patients	24 (4M 20F). 12 (**PMED**) - 12 chronic.	27 ± 11	(S) Thermal -heat-: thermode blockTMJ therapy	SCR and SCL (median, SD, min, max, 1st and 2nd derivative)	VAS	Badura et al., 2024^109^
**EEG**	Acute	Patients	30 (13M 17F)	29-73.	(S) Laser Pulse	Mean IPI variability, LEP latency and amplitude,	NCS-R	Naro et al., 2017^77^
		Volunteers	9 (7M 2F)	Not provided	(S) Mechanical: hand pressure application	Delta, Theta, Alpha, low Gamma and Beta (decomposed into 4 bands).	NRS	Okolo & Omurtag, 2018^71^
		Volunteers	62 (30M 32F)	Young: 22.95 ± 3.58. Old: 68 ± 6.	(S) Thermal -heat-: hot air.	Delta, Theta, Alpha, low Gamma, high Gamma and Beta bands. GBO	VAS	Zhou et al., 2020^72^
		Volunteers	13 (8M 5F)	20-52	(S) Electrical: TENS	HFD-based (HFD, HFD_ACF, HFD_VAR), GP-based (GP, GP_ACF, GP_VAR), Correlation-based (HFD, HFD_ACF, GP_ACF), VAR-based (GP, HFD_VAR, GP_VAR)	N/A	Tripanpitak et al., 2020^75^
		Volunteers	20 (17M 3F)	27.4 ± 5.7	(S) Thermal -cold-: CPT	Delta, Theta, Alpha, low Gamma, and Beta bands.	VAS	Wang et al., 2020^74^
		Volunteers	32 (12M 20F)	19-35	(S) Thermal -cold-: CPT	Delta, Theta, Alpha, low Gamma, and Beta bands.	NRS	Yu et al., 2020^99^
		Volunteers	20 (11M 9F)	23-42	(S) Thermal -cold-: CPT	Delta, Theta, Alpha, Beta, and low Gamma bands.	NRS	Yu et al., 2020^94^
		Volunteers	24 (17M 7F)	21	(S) Thermal -cold-: CPT	Spatial spectral-temporal signal analysis	VAS	Wu et al., 2022^110^
		Volunteers	36 (14M 22F)	25.36 (20-56)	(S) Thermal -heat-: hot water	Alpha band (peak, individual alpha frequency) with closed and open eyes	VAS	Valentini et al., 2022^111^
		Volunteers	28 (10M 18F)	29.23 ± 13.83	(S) Topical capsaicin. (T) Electrical: rTMS, AiTBS	Delta, Theta, Alpha, Beta, and low Gamma bands.	VAS	Tan et al., 2024^112^
		Volunteers	12 (12F)	20.4 ± 0.9	(S) Electrical: electrodes.	Evoked Potentials amplitude and latency.	NRS, QST	Chen et al., 2023^113^
		Volunteers	80 (46M 34F)	25.2 ± 2.06	(S) Thermal -cold-: CPT. (T) Electrical: TENS, tDCS.	Spectral power density analysis in Alpha, Delta, Theta, Beta and Gamma bands.	NRS	Mujib et al., (2024)^114^
		Patients	33 (21M 12F)	7.41 ± 3.38	(S) Mechanical: Arterial puncture.	Pre-processed signal.	N/A	Fu et al., (2024)^115^
		Volunteers / Patients	86 (49M 57F)	27.9 ± 1.9 weeks	(S) Mechanical: skin breaking procedures done in NICU	Somatosensory Evoked Potentials waves and scores.	N/A	Coviello et al., 2024^116^
	Chronic: joint, widespread, back, neuropathic.	Volunteers /Patients	**Patients**. 101 (32M 69F). **Control**. 88 (28M 60 F).	**Patients**. 58.1 ± 13.6. **Control**. 57.5 ± 14.2.	Patients vs control group	Signal global field power peaks.	painDETECT / PDI	May et al., 2021^117^
	Chronic: LBP	Patients	27 (7M 20F)	44.6 ± 2.3	Chronic pain resting state	ABO (eyes closed, eyes open and normalised).	VAS	Feng et al., 2021^76^
	Chronic: posttraumatic headache	Volunteers / Patients	**Patients**. 26 (21M 5F). **Control**. 20 healthy (13M 7F)	**Patients**. 41.9 ± 11.1. **Control**. 40.7 ± 15.5	Evoked potentials	Evoked Potentials amplitude	VAS	Jessen et al., 2024^118^
	Chronic: Cancer related pain	Patients	3	55-75	(T) Music Therapy	Delta, Theta, Alpha, low Gamma, and Beta bands.	NRS	Hunt et al., 2021^119^
	Chronic: osteoarthritis, lower back pain	Patients	4 (1M 3F)	85-92	Chronic pain resting state	Detrended fluctuation analysis of the signal.	PAINAD scale	Pu et al., 2021^120^
	Chronic: trigeminal and postherpetic neuralgia, spinal cord injury …	Volunteers / Patients	211 (101M 110F)	18-85	Chronic pain resting state	Pi.	VAS / NRS	An et al., 2017^73^
**fNIRS**	Acute	Volunteers	30 (23M 7F)	31.7 ± 8.7	(T) Electrical: TENS	10 statistical features from ΔHbO2 and ΔHHB each	NRS	Khan et al., 2024^80^
	Chronic: cervical shoulder syndrome	Patients	20 (20M)	36.6 ± 7.2	(S) Mechanical: Pressure application (T) Electrical: TENS	HbO	VAS	Du et al., 2023^78^
	Chronic: Osteoarthritis	Patients	30	50–85	(T) Neuromodulation: tDCS+MBM(S) Thermal -heat-: thermode block	HbO	NRS	Pollonini et al., 2020^79^
**ECG, EDA**	Acute	Volunteers	BioVid Database (Part A – 67 subj.)	Not provided	(S) Thermal -heat-: thermode block	Whole pre-processed signal	N/A	Subramaniam & Dass, 2021^100^
		Volunteers	30 (15M 15F)	34 ± 11.9	(S) Thermal -heat-. Electrical	**EDA**. Max, average, AUC and SD of SCR and SCL from cvxEDA. Phasic driver (average rise time, number of peaks, max peak amplitude). **ECG**. HR (mean, median), HRV (SDNN, RMSSD, LF).	N/A	Jiang et al., 2024^36^
		Volunteers	BioVid Database (Part C - 87 subj.)	Not provided	(S) Thermal -heat-: thermode block	**EDA**. 11 statistical features. **ECG**. HR, HRV (SDNN, RMSSD)	N/A	Jiang et al., 2024^101^
	Chronic: Cancer-related pain	Patients	**Case1**. 1 F. **Case2**. 1 M.	**Case1**. 66. **Case2**. 74	(T) Opioids. Neuromodulation: PENS and HF.	**EDA**. SCR (latency, sum, average) ISCR. **ECG**. RR (mean, SD).	NRS	Cascella et al., 2023^32^
	Chronic: cancer-related pain	Patients	64	60.6 ± 13.3	(T) Pharmacologic: Opioids, pain adjuvants	**EDA**. Mean amplitude of CDA and TTP. **ECG**. HR, HRV (RRI, SDNN)	NRS	Cascella et al., 2024^28^
**ECG, Face Exp**	Acute	Volunteers	BioVid Database (Part A – 87 subj)	Not provided	(S) Thermal -heat-: thermode block	Whole pre-processed signal	N/A	Gkikas et al., 2024^121^
**ECG, PPG**	Acute	Volunteers	20 (10M 10F)	26.5 ± 6.4	(S) Thermal -heat-: thermode block	PTT, PPGA	NRS	N van Velzen et al., 2015^45^
		Volunteers	**Trial**. 30 (14M 16F). **C**. 10 (7M 3F)	**Trial**. 22.5 ± 1.9. **C**. 22.5 ± 1.3	(S) Thermal -heat-: water	**ECG**. RRI, HR, HRV. **PPG**. PPGA, BL, ANSS	NRS	Ye et al., 2017^52^
		Volunteers	25 (11M 14F). 22.44 ± 1.85 years.	22.44 ± 1.85	(S) Thermal -heat-: water	**PPG**. PPGA, BL, ANNS. **ECG**. HRV (RRI, HF, LF, LF/HF, SDNN)	NRS	Jhang et al., 2021^31^
	Chronic: cervical, lumbar	Patients	66 (23M 43F)	62.86 ± 12.10	(T) Electrical: RFT	**ECG**. HR, RRI, short HRV (LF, HF, VLF, normalised LF, normalised HF, LF/HF). **PPG**. PPGA, BL, PPI, ANSS, SSI.	VAS	Ye et al., 2017^29^
	Chronic: Myofascial Pain Syndrome	Patients	37 (10M 27F)	41.84 ± 13.68	(T) Electrical &Thermal -heat-: thermotherapy	HRV (**ECG**) and PRV (**PPG**): HR, mean RRI, SDNN, RMSSD, VLF, LF, HF, LF/HF. **PPG**: PPGA, SPI, ANSS, ANSSi, BL	VAS	Ye et al., 2018^30^
	Intraoperative / post-operative	Patients	40 (34M 6F)	36.86 - 37.86 weeks	Surgery: open and laparoscopic inguinal hernia repair.(T) Analgesics	NIPE Monitor Index	N/A	Sakthivel et al., 2024^17^
		Patients	78 (31M, 47F)	51.8 ± 10.9	(T) Pharmacologic: Opioids	60 morphological, spatiotemporal, interval and dynamic feat.	VAS	Seok et al., 2019^84^
		Patients	192 (84M 108F)	54 ± 12	Pre vs postsurgical pain	**PPG**. SPI. **EEG**. ANI	NRS	Lee et al., 2019^34^
**ECG, PPG, BP**	Acute	Volunteers	11 M	22.8 ± 1.7	(S) Electrical	**BP, PPG** (PPGA). **PPG/ECG/BP**. Arterial stiffness index	NRS	Matsubara et al., 2018^49^
	Post-operative	Patients	30 (19M 11F).	42 ± 5	(T) Pharmacologic: sufentanil	**PPG**. AC, DC, AC/DC. **BP**.SBP, DBP, MAP. **ECG**. Heart rate.	VAS	Ling et al., 2014^41^
**ECG, PPG, EDA**	Acute	Volunteers	6 (4M 2F)	22-25 ± 3	(S) Electrical	12 statistical feat.: means; SD; means of the abs values of the first diﬀerences and the norm signals; means of the abs values of the second diﬀerences and the norm signals; min and max values; min and max ratios; range; median.	VAS	Chu et al., 2017^85^
**ECG, PPG, fMRI**	Acute	Volunteers	**EXP1**. 30 (30M). **EXP2**. 22 (22M).	**EXP1**. 23.5 ± 1.4**EXP2**. 22.7 ± 1.0	(S) Electrical	Arterial Stiffness	NRS and VAS	Tsuji et al., 2021^50^
**EDA, Face Exp**	Chronic: PIFP	Patients	**Trial**. 67. **Control**. 28.	Not Provided	Trial vs control group		VAS	Rokicki et al., 2022^122^
	Post-operative	Patients	45 (31M 14F)	5-17	(S) Mechanical: manual abdominal pressure	**EDA**. TSD triangular matrix (mean, SD, entropy). SCL (SD)	NRS	Susam et al., 2022^92^
**EDA, Self-Assessment**	Intraoperative/ post-operative	Patients	15 (4M 11F)	63.73 ± 7.85	Pre vs postsurgical pain	**EDA**. SCR	NRS	MacNeill & Mayich (2020)^123^
**EEG, ECG, Resp**	Acute	Volunteers	18 (8M 10F)	27.22 ± 4.03	(S) Electrical. (T) Virtual reality hypnosis	**EEG**. Evoked potentials and time-frequency responses. **ECG**. HRV (RRI, HR, RMSSD). **Resp**.	VAS	Rousseaux et al., (2023)^124^
**EMG, ECG, EDA**	Acute	Volunteers	BioVid Database	Not Provided	(S) Thermal -heat-: thermode block	Amplitude, freq, stationarity, entropy, linearity, and variability derived features from **EMG** (Zygomaticus, corrugator and trapezius muscle), **EDA** (SC), **ECG** (HR, interbeat interval, HRV)	N/A	Walter et al., 2014^24^ Gruss et al., 2015^25^
		Volunteers	BioVid Database	Not Provided	(S) Thermal -heat-: thermode block	Whole pre-processed signal	N/A	Thiam et al., 2019^98^
		Volunteers	BioVid Database	Not Provided	(S) Thermal -heat-: thermode block	22 time-series feat. (each): distribution, simple temporal statistics, linear and nonlinear autocorrelation, successive differences, fluctuation analysis.	N/A	Pouromran et al., 2021^67^
		Volunteers	BioVid Database (Part B - 86 subj.)	Not Provided	(S) Thermal -heat-: thermode block	155 feat. including amplitude, variability, stationarity, entropy, linearity, similarity and freq.	N/A	Albahdal et al., 2024^87^
**EMG, ECG, EDA, Face Expr, Audio**	Acute	Volunteers	X-ITE Database (127 subj.)	18-50	(S) Electrical. Thermal -heat-: thermode block	Time-series statistic descriptor of the signals (1st & 2nd derivative, min, max, mean, SD).	VAS	Othman et al., 2022^95^ Othman et al., 2023^96^
	Acute	Volunteers	X-ITE Database (127 subj.)	18-50	(S) Electrical. Thermal -heat-: thermode block	Whole pre-processed signal.	VAS	Gruss et al., 2019^21^
**ECG, PPG, EDA, Resp**	Acute	Volunteers /Patients	**Volunteers**. 52 (17M 35F). **Patients**. 20 (5M 15F).	**Volunteers**. 27.4 ± 6.6. **Patients**. 40.9 ± 14.4	(S) Thermal -heat-: thermode block	**ECG** (Mean, SDNN, RRI slope, RMSSD, number of R peaks), **Resp** (Number & mean amplitude of phases), **PPG (**PR)., **EDA** (SCR, SCL, dPhEDA, TVSymp). Max, min, range, SD, interquartile range, mean, local max and min for all.	VAS	Khan et al., 2023^82^
**EMG, ECG, PPG, EEG, Face Exp**	Acute	Patients	109 (56M 53F)	4 (1–23) days	(S) Mechanical: heel lances	Reflex withdrawal, HR, SPO2, facial expression responses, noxious-evoked brain activity.	N/A	Van der Vaart., 2019^86^
**EMG, EDA**	Acute	Volunteers	BioVid Database	Not Provided	(S) Thermal -heat-: thermode block	**EMG** (zygomaticus). FF, RMS, ZCD. **EDA**. SCR (slope)	N/A	Sen & Pal, 2021^125^
**EMG, PPG, EDA, EEG, BP, Resp, Face Exp**	Acute	Volunteers	26 (7M 19F)	20.7 ± 2.4	(S) Thermal -cold-: CPT	**EEG**. Frequency bands (delta, theta, alpha, beta, gamma), GD, EB, HEM, VEM. **EDA** (SC), **PPG** (PR)**, BP** (Diastolic, systolic), **Pupillometry** (Pupil Dilation)	NRS	Lin et al., 2022^23^
**PPG, EDA, Resp**	Acute	Volunteers	22 (10M 12F)	27 ± 4.19	(S) Electrical: TENS	**PPG**. Rate (mean, amplitude), PRV (time, freq, nonlinear domain feat.). **EDA**. SCR (number of peaks, max, amplitude (mean, median, SD, max, min, range, interquartile range metrics). **Resp**. Rate variability, sinus arrhythmia, amplitude (min, max, mean), rate (min, mean, max, min and max time).	VAS	Fernandez-Rojas et al., 2023^57^
**PPG, EDA, Temp, Acc**	Acute / Chronic: Upper limb CRPS	Patients	20 (8M 12F)	48.4 ± 9.2	(S) Mechanical: physiotherapy.	NOL Index	NRS	Santella et al., 2022^16^
	Post-operative / Acute	Patients	54 (39M 15F)	65.76 ± 11.77	(S) Mechanical: non-invasive BP, chest tube removal	NOL Index	NRS	Gélinas et al., 2021^15^
**PPG, EEG**	Post-operative	Patients	98 (50M 48F)	62.9 ± 12.8	Pre vs postsurgical pain	**PPG**. PI. **EEG**. BIS	VAS	Kwon et al., 2019^51^
**Pupillometry**	Post-operative / Chronic	Patients	18 (4M 14F)	21-72	(T) Pharmacologic: analgesics. Surgery	PUAL	NRS	Behrends & Larson., 2024^83^

The table is organized and grouped by signals used and type of pain assessed. In “Pain Modulation Method”, (T) is used to denote a pain treatment approach while (S) refers to the pain stimulation approach. Only features related to physiological signals were reported under the “Physiological Markers Studied” column. “Age” provided is in years unless stated otherwise.

The BioVid Heat pain database is the first publicly accessible database that combines physiological parameters, video signals, and stimulus intensity for studying acute pain in healthy subjects. The database was first introduced in 2013, and it was publicly released for use in non-commercial research in 2018. It contains data from 90 subjects divided into three age groups of 30 subjects each: 18–35, 36–50, and 51–65. All groups were equally divided between male and female participants. The data were acquired from an experimental study where pain was induced using thermal stimulation from a thermode block placed on the right arm, which produces heat on the skin. The physiological signals recorded include electrocardiography (ECG), electromyography (EMG), electroencephalography (EEG), and electrodermal activity (EDA). The video signals were recorded to capture participants’ facial expressions during the experimental pain induction. The database is available for non-commercial research upon request^20^.

On the other hand, the X-ITE Pain Database is a publicly available multimodal dataset designed to support research in automatic pain recognition. It was collected from 134 participants through controlled experimental pain induction (heat and electrical stimuli) at multiple intensities, including both phasic and tonic protocols. The dataset comprises approximately 24,000 phasic and 800 tonic stimuli, along with their associated responses. A wide range of modalities were recorded, including facial video (frontal, side-view, and thermal), body video (RGB and depth), audio, as well as physiological signals such as electrodermal activity (EDA), electrocardiogram (ECG), and surface electromyography (sEMG) from several muscle sites. This breadth makes the X-ITE database particularly valuable for multimodal approaches that combine behavioural and physiological data in the context of pain assessment. The database is available for research upon request^21^.

Recently, a new database called PainMonit was also released. This database has two datasets that include not only ECG, EMG, and EDA signals but also photoplethysmography (PPG), temperature (Temp) and respiration (Resp) signals for the study of pain. The first dataset, the PainMonit Experimental Dataset (PMED), represents experimental data collected from 55 healthy subjects, comprising 22 males and 33 females, with an average age of 27.47 ± 6.90 years. These subjects underwent thermal (heat) pain elicitation from a thermode block. In addition to information about stimulus intensity, like the BioVid database, this dataset provides self-reported pain intensity VAS score from the subjects acquired over the duration of the experiment^22^.

The second dataset includes a clinical dataset, PainMonit Clinical Dataset (PMCD), acquired from 49 subjects (21 males and 28 females) with an average age of 27.47 ± 6.90 years, diagnosed with chronic arm and neck pain. Signals were collected before, during and after a facial physiotherapy session that was prescribed for their pain management. Besides the signals, self-reporting NRS scores are included in the dataset information^22^.

### Study heterogeneity and reporting indicators

To provide an overview of study characteristics and facilitate the interpretation of results, key features of the included studies were summarised in a heterogeneity table (Table 2). The table presents information such as the type of pain assessed, the study population, the sample size, sex distribution, methodological approach, measure used in comparative analysis, and whether data were derived from public databases. This summary allows readers to appreciate the diversity across studies in terms of design, population, and measured outcomes, highlighting potential sources of variability in the literature.

**Table 2:** Analysis of heterogeneity across all included studies

Characteristics	Categories (*n*, %n)
Type of pain*	Acute (57, 64%), chronic (18, 20.2%), intraoperative/postoperative (18, 20.2%)
Study population	Volunteers (45, 50.6%), patients (38, 42.7%), mixed (6, 6.7%)
Sample size	<20 (12, 13,5%), 20-50 (37, 41.6%), >50 (34, 38.2%), Not reported (6, 6.7%)
Sex	Balanced (19, 21.3%), majority male (15, 16.9%), majority female (27, 30.3%), only female (3, 3.4%), only male (4, 4.5%), not reported (21, 23.6%)
Methodological approach *	Pain induction (59, 66.3%), pain modulation (22, 24.7%), observational (7, 7.9%), surgery (10.1%)
Public Database	Yes (13, 14.6%), No (76, 85.4%)

For characteristics marked with (*), the total percentage may exceed 100% as individual studies may report multiple attributes within a single category.

The studies demonstrated substantial heterogeneity across key characteristics. Most addressed acute pain (64%), with fewer focusing on chronic or intraoperative/postoperative pain. Study populations were nearly balanced between volunteers and patients, with some using mixed groups. Sample sizes varied widely, though most ranged between 20 and 50 participants, followed by studies with more than 50 participants and only a few reporting fewer than 20 subjects. Sex distribution was inconsistent, with balanced samples in only 21.3% of studies, while most showed either male or female predominance, and nearly a quarter did not report sex at all. Methodological approaches were diverse, dominated by pain induction (66.3%), followed by pain modulation, observational, and surgical studies. Public database use was limited, with only 14.6% of studies employing shared datasets.

### Physiological responses to pain

This section explores the physiological signals linked to variations in pain perception, as reported in the reviewed literature. Table 1 summarises the extracted data, organised by signal type and further categorised by the type of pain studied to support a clear presentation of evidence. Each physiological signal identified as a potential pain marker is discussed in turn, highlighting features that have been previously investigated as possible biomarkers of autonomic nervous system activity and pain sensation. The section also explores how these features behave across different pain contexts, including acute, chronic, and perioperative/postoperative pain.

### Electromyography (EMG)

Electromyography is defined as the measurement of muscle electrical impulses generated when the muscle fibres contract. EMG signals can be measured using an intramuscular needle or electrodes placed on the skin’s surface^23^. The signal’s potential for pain evaluation relies on the idea that electrical muscle activity indicates general psychophysiological stimulation. Higher muscle tone is linked to increased sympathetic nervous system activity, while a decrease in electrical muscle activity is associated with parasympathetic activity^24^.

Several studies have been conducted using the BioVid Heat Pain Database. Amplitude and similarity-derived parameters measured from the zygomaticus and corrugator muscles have demonstrated a significant correlation with thermal stimulation levels (4 levels) in healthy subjects^24,25^. Additionally, these parameters exhibit high accuracy, sensitivity and specificity to differentiate between no stimulus vs painful stimulation when used to train SVM models^25^.

In contrast, statistical features extracted from the database’s EMG signals, such as the ratio of mobility of the first derivative of the signal (FF), root mean square value (RMS), and zero crossing, or the number of times the pattern crosses the zero-potential line (ZCD), showed no significant correlation with different acute pain states in healthy subjects^23^.

Jurth et al. (2014) investigated the reliability of the *Nociceptive Flexion Reflex (NFR)* and self-assessment for quantifying pain through test-retest measures using a Conditioned Pain Modulation (CPM) approach^26^. NFR refers to a leg withdrawal reflex measured in the biceps femoris muscle after stimulation of the sural nerve at the foot. The study found no statistically significant correlation between this parameter and pain stimulation/reduction.

### Electrocardiography (ECG)

Electrocardiography is a technique used to measure the electrical activity of the heart^7^. The most commonly used feature in pain research is *Heart Rate Variability (HRV)*. HRV is an indicator of the variability in time between heartbeats that has been linked to cardiac autonomic regulation^27^. This measure contains time and frequency domain parameters with different information regarding the ANS.

Time-domain parameters such as Heart Rate (HR) and R-R Interval (RRI) are commonly used indicators of autonomic nervous system activity. HR represents the number of heartbeats per minute, reflecting the frequency of cardiac cycles. The RRI refers to the time elapsed between two consecutive heartbeats, measured as the interval between R-waves on the ECG^28^. These parameters reflect the dynamic balance between the two branches of the ANS: the parasympathetic and sympathetic nervous systems. Parasympathetic influence on HR is primarily mediated by the vagus nerve through the release of acetylcholine, which acts to slow the heart rate^29^.

With greater parasympathetic (vagal) activity, HR tends to decrease and RRI increases, indicating a relaxed state. Conversely, a shorter R-R Interval and elevated heart rate are often associated with increased sympathetic activity, which prepares the body for ‘fight or flight’ responses^29^. Studies have reported a significant positive correlation between HR and chronic pain intensity^29,30^.

On the other hand, regarding the RRI marker, research has demonstrated a significant negative correlation with chronic pain intensity^29,30^ and acute pain detection in healthy volunteers^31^. Similarly, the *R-R slope* indicates a strong correlation with acute pain levels in healthy volunteers^24^ while *R-R standard deviation (RRSD)* shows a significant positive correlation with pain intensity in cancer patients experiencing chronic pain^32^.

Other time domain parameters studied as potential pain biomarkers include the *Standard Deviation of the Normal-to-Normal Beat Interval (SDNN)* and the *Square Root of the Mean Squared Differences of Successive NN intervals (RMSSD)*. Research has reported that SDNN has a negative correlation with chronic pain intensity^30^ but a significant positive correlation with acute pain in healthy volunteers^31^. Similarly, RMSSD has reported a negative correlation with chronic pain intensity^30^.

Also, HRV frequency domain parameters, which measure the distribution of power across different frequency bands in the heart rate signal, have been shown to be impacted by pain. *High Frequency (HF)*, between 0.15 and 0.4 Hz, is affected by vagal parasympathetic activity via the release of acetylcholine by the vagus nerve^33^ and has shown a significant negative correlation with chronic pain intensity^29,30^ and acute pain in healthy volunteers^31^. In contrast, Lee et al. (2019) found that HF has described a negative correlation with postoperative pain. They also suggested that this marker itself might not be sufficient to distinguish moderate pain states^34^.

*Low Frequency (LF)*, ranging from 0.04 to 0.15 Hz, reflects both sympathetic and vagal activity. Research shows a significant negative correlation between this parameter and chronic pain intensity^29^ and acute pain in healthy volunteers^31^. Similarly, *Very Low Frequency (VLF)*, between 0.003 and 0.04 Hz, also reports a negative correlation with chronic pain intensity^29^.

Furthermore, the Analgesia Nociception Index (ANI) has been introduced to evaluate acute nociception. This index is derived from the RR and a frequency domain analysis of the HF component. Initially suggested for monitoring pain nociception during surgery, studies have demonstrated that it also negatively correlates with self-reported pain in postoperative patients^35^.

To improve the pain recognition capabilities of physiological signals, Jiang et al. (2024) proposed two methods for representing individual pain sensitivity using the HRV features SDNN, RMSSD and LF at resting state. These methods are named SensPredict and SensHRV and calculate a pain sensitivity score using linear regression and neural networks, respectively^36^.

Despite the evidence previously discussed, the reliability of HRV features for assessing pain remains uncertain. While some research suggests a link between self-reported pain and HRV features, other studies have reported a lack of correlation between these parameters in chronic pain scenarios, indicating that they can’t be used as surrogate biomarkers of pain^28,37^.

For example, the *Low-to-high Frequency Ratio (LF/HF)* parameter, hypothesised to represent the sympathetic and parasympathetic balance index^33^, has shown a strong positive correlation with chronic pain intensity^30^. Despite this, its accuracy in terms of sympathetic activity has been debated^38^. Particularly, Umeda & Okifuji (2022) discovered that, although there is a correlation with severe self-reported acute pain scores, the log-transformed LF and HF features do not correlate with moderate acute pain levels^39^.

Furthermore, Tracy et al. (2018) found that resting LF and HF features positively correlate with pain threshold in healthy subjects. However, they suggested that there are sex differences that need to be considered in HRV marker responses to pain^40^. Ling et al. (2014) particularly addressed the limitations of using HRV parameters in individuals taking antihypertensive agents, such as β-blockers. These medications can suppress the expected increase in heart rate and blood pressure in response to pain stimuli. In their study, they found no correlation between HRV markers and the reported pain intensity in postoperative patients^41^.

### Photoplethysmography (PPG)

Photoplethysmography is a non-invasive optical technique that measures changes in light absorption or transmission caused by the volumetric changes in pulsating blood associated with the cardiac cycle. As a result, the PPG waveform is generated from light that is not absorbed by the irradiated tissue but rather scattered. This reflected or transmitted light is detected by an optical sensor, which processes it to indicate light absorbance. The intensity of light recorded is inversely proportional to the amount of light absorbed by the tissue^42^.

PPG’s potential use as a pain biomarker comes from its ability to monitor changes in the peripheral vascular system caused by pain. Noxious stimuli generating pain affect the peripheral vascular system by activating the sympathetic nervous system (SNS), a key regulator of the cardiovascular system that controls cardiac output and peripheral vascular resistance^43,44^. This activation induces vasoconstriction, reducing arterial compliance, a measure of arterial elasticity, and narrowing vessel diameter^45^. Those vascular changes associated with the pain sensation can be monitored through the analysis of the photoplethysmography signal^46^.

**Fig. 3:**
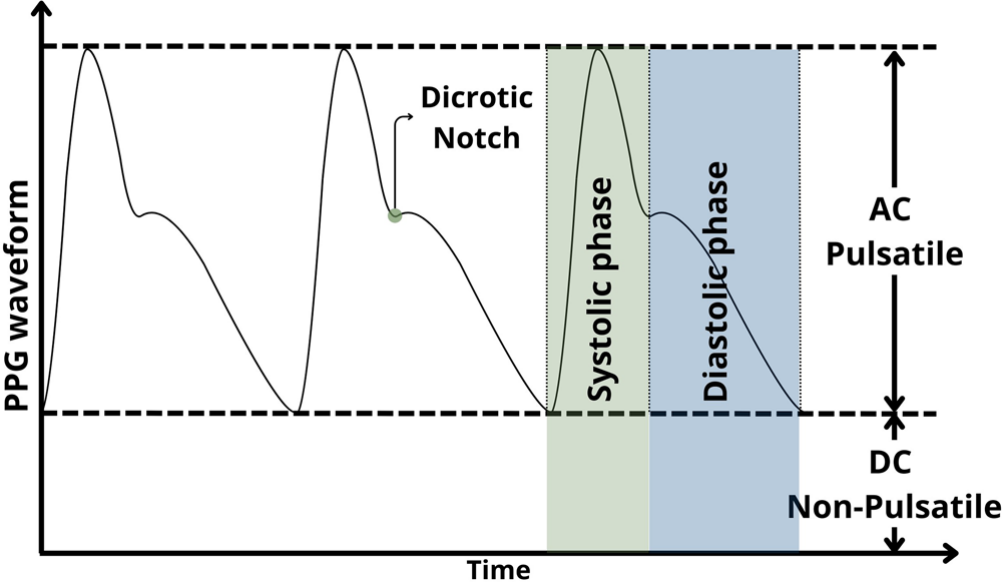
Schematic representation of the PPG waveform. The figure shows a schematic of the characteristic morphology of a photoplethysmography waveform.

PPG signal can be analysed using a morphological approach. As illustrated in Fig. 3, the PPG signal can be decomposed into two major components associated with the absorbance of the pulsatile vs non-pulsatile tissue components: the AC and DC waves. The non-pulsatile wave *(DC)*, or *Baseline (BL)*, comes from the relatively constant light absorbed by the non-pulsatile tissue components. It is inversely proportional to the blood volume in the tissue^41,42^. It’s been reported to have a strong positive correlation with chronic pain^30^, acute pain in healthy subjects^31^ and postoperative pain^47^.

The pulsatile wave (AC) represents the arterial blood component that is synchronised with the heartbeat^41,42^. One of the most straightforward morphological features that can be extracted from the AC component is the *PPG Amplitude (PPGA) –* also referred to as *Systolic Peak Amplitude (SPA)*, AC Amplitude from previous diastole (ACAdia), and pulse height. This amplitude has been shown to have an inverse correlation with sympathetic and vasoconstrictor nerve tone^30^. In addition, the ratio of the PPGA to the DC signal’s amplitude is defined as the *AC/DC ratio* or *Perfusion Index (PI)*, which reflects changes in tissue microcirculation^13^.

Another parameter that can be extracted is the *Pulse-Pulse Interval (PPI)*, also called *Heart-Beat Interval (HBI)*, representing the time elapsed between successive pulses. The PPI can be systolic *(PPIsys)* if the interval is measured between systolic peaks or diastolic *(PPIdia)* if it’s calculated between diastolic peaks^47^. A negative correlation between this parameter and sympathetic tone has previously been reported^30^, as well as a significant negative correlation with chronic pain intensity^29,30^. However, no significant changes have been found between the diastolic and systolic components regarding postoperative pain^47^.

Paloheimo et al. (2010)^48^ introduced a numerical descriptor of the autonomic nervous system that uses the PPI and PPGA features to calculate the index, called *Autonomic Nervous System State (ANSS)*. Equation 1 shows the formula to calculate this indicator, which has shown a negative correlation with sympathetic tone^30^.

1 $${ANNSS}={PPGA}* {PPI},\, \% s$$

Equation 1*The equation to calculate the ANSS. The analysis is made pulse-by-pulse*.

Overall, researchers have reported a significant negative correlation between acute pain intensity in healthy volunteers and PPGA^31,45,49,50^, and ANNS^31,45^. This indicates that as pain intensity increases, those variables tend to decrease. This negative correlation is also reported between postoperative pain intensity and PPGA^41,47^, ANNS, and AC/DC ratio^41^.

This behaviour may be linked to the excitability of the sympathetic nerves, which leads to a decrease in peripheral perfusion. This way, increased pain intensity could reduce the AC wave amplitude and the AC/DC value. In contrast to those outcomes, Kwon et al. (2019) found no significant correlation between postoperative pain and perfusion index or AC/DC ratio^51^.

On the other hand, Ye et al. (2017) found that, in acute pain scenarios, the negative correlation among the exposed features switches after a few minutes of applying the noxious stimuli, showing a positive correlation between pain intensity and PPGA/ANNS features associated with prolonged pain stimulation^52^. This trend is similar to the one reported in studies conducted on chronic pain patients undergoing radiofrequency therapy^29^, electrotherapy and thermotherapy^30^ for pain relief, where they found a significant decrease in PPGA and ANSS associated with chronic pain intensity reduction.

Another index developed from the PPGA and PPI features is the *Surgical Pleth Index (SPI)*, formerly known as *the Surgical Stress Index (SSI)*. It is a tool used to evaluate the effectiveness of intraoperative analgesia during general anaesthesia. It is particularly useful for detecting nociceptive events when administering total intravenous anaesthesia. The SPI values range from 0 to 100, reflecting the body’s level of surgical stress^13,29^. Equation 2 shows the equation used to calculate the SPI level, where ${{\rm{PPI}}}_{{\rm{norm}}}$ and ${{\rm{PPGA}}}_{{\rm{norm}}}$ denote the normalised values of the features.

2 $${SPI}=100-\left(0.33* {{PPI}}_{{norm}}+0.67* {{PPGA}}_{{norm}}\right)$$

Equation 2*The equation to calculate the SPI.*

Generally, studies have found statistically significant differences between pain and no pain states in studies conducted on chronic pain^30^, and postoperative pain^34^ patients. Nevertheless, the effectiveness of this parameter has been discussed since it has displayed notable interindividual variability compared to others, like ANI. Additionally, SPI alone has proven insufficient to distinguish moderate pain states^34^.

Yang et al. (2018) analysed morphological parameters that can be extracted from the PPG signal waveform. This study identified specific correlation patterns among some of the parameter groups and postoperative pain including: negative correlation with area-based parameters - PA, Area of a Systolic Phase (Asys), Area of a Diastolic Phase (Adia), Triangular Area of a Pulse (TriA), Triangular Area of a Systolic Phase (TriAsys), and Triangular Area of a Systolic Phase (TriAdia)-; a negative correlation with amplitude-based parameters -AC Amplitude from baseline (ACAbl)-; a negative correlation with slope-based parameters – Rising Slope (RS), Rising Slope Length (Lrs), Maximum Rising Slope (RSmax), Failing Slope (FS), and Failing Slope Length (Lfs)-^47^.

Researchers have also analysed combinations of morphological parameters in binary classification scenarios, finding that systolic peak variation (SYSvar, ACVsys/ACAdia), diastolic peak variation (DIAvar, ACVdia/ACAdia), PPI variation (HBIvar), ACAdia, ACVsys/ACAbl, ACVdia/ACAbl showed the best individual performance among all the combinations analysed^47,53^. In contrast, when trying to distinguish between three different pain levels, the best performance has been shown by median Atotal/ACAbl, max Asys/ACAbl, median Asys/ACAbl, median Adia/ACAbl, median (Atotal/ACAbl)/Lsys, median (Atotal/ACAbl)/Ldia, median (Atotal/ACAbl)/PPIonset, median (Asys/ACAbl)/PPIonset, median (Asys/ACAbl)/Ldia, median (Adia/ACAbl)/Lsys, median (Adia/ACAbl)/Ldia^54^.

Furthermore, Cho et al. (2018) introduced the *Nasal Photopletismography Index (NPI)*, built based on the DIAvar and the PPIvar parameters. It uses a PPG sensor placed between the columella and the nasal septum. A value close to 100 represents severe pain, and 0 means no pain. They used this novel index in a study trying to assess postoperative pain intensity, showing a higher accuracy (75.3%) when compared to the SPI index (64.8%), whose PPG signals are acquired from a PPG finger probe^53^.

3 $${NPI}=462.43* {{DIA}}_{var}-683.11* {{HBI}}_{var}+35.55$$

Equation 3*Mathematical expression of the Nasal Photoplethysmography Index (NPI)*.

*Pulse Rate Variability (PRV)*, a measurement of the changes in pulse rate (PR) over time, has been widely used in the last few years as a surrogate measure of *Heart Rate Variability (HRV)*. Researchers often use both terms interchangeably, particularly when discussing measurements derived from the PPG waveform. However, a recent study has shown that PRV and HRV do not always provide the same information about autonomic activity, for example, under extreme weather conditions^55^. Therefore, this paper distinguishes the two measures based on the specific signals used for their calculations, regardless of the terminology employed in the original studies reviewed.

The features derived from HRV – See 4.2. Electrocardiography (ECG) - can also be calculated from PRV; however, PRV features are based on pulse rate signals rather than direct heart rate signals. Studies utilising time-domain parameters have revealed several key findings. Firstly, studies analysing *Pulse Rate (PR)* have found a strong positive correlation between this variable and chronic pain intensity^30^, but no significant correlation with acute pain in healthy volunteers^23^. *Standard Deviation of the Normal-to-Normal Beat Interval (SDNN)* and *Square Root of the Mean Squared Differences of Successive NN intervals (RMSSD)* have been associated with a positive statistical correlation to chronic pain intensity^30^. However, it’s been reported that RMSSD does not hold a significant correlation with postoperative pain^56^. Furthermore, the *Average of the Peak-to-Peak intervals (AVNN)* has shown a strong correlation with postoperative pain^56^. Finally, in terms of the PRV frequency domain parameters, *High Frequency (HF), Low Frequency (LF) and Very Low Frequency (VLF*) all reveal a significant negative correlation with chronic pain intensity^29^.

Ultimately, other features previously studied in pain assessment research include time domain features such as mean (M), variance (V), skewness (S), kurtosis (K), crest factor (CF), shape factor (SF), impulse factor (IF), margin factor (MF), Shannon energy (SE), log energy (LE), mobility (Mob), and complexity (Comp); and frequency domain features, like spectral flux (SpF), spectral crest (SpC), spectral flatness (SpFt), spectral centroid (SpCent), spectral kurtosis (SpK), spectral spread (SpSp), spectral roll-off (SpR), spectral slope (SpS), spectral decrease (SpD), spectral entropy (SpE), and mean frequency (SpM)^23,57^. Other time domain features with a potentially strong correlation include the pulse’s Rise Time (the average of the time between a valley and the next peak) and Fall Time (the average of the time between the peak and the next valley)^56^.

### Electrodermal Activity (EDA)

Previously known as Galvanic Skin Response (GSR), EDA is a measurement of the electrical activity on the skin in response to sweat secretion. The reasoning behind this approach, as a potential signal for pain assessment, is that sweat glands are controlled by the sympathetic nervous system, with no contribution from the parasympathetic nervous system. Consequently, when a person experiences acute pain that activates the sympathetic nerve, it can affect the electrogalvanic properties of the skin due to changes in the eccrine sweat glands^24,41^.

Two methods are used for EDA measurements, based on measuring the electrical difference between two electrodes placed on the skin. In the exosomatic technique, a small electrical current from one of the electrodes is applied to the skin, and the resistance is then measured^58^. This is the most commonly used method, retrieving the *Skin Conductance (SC)*, the reciprocal of skin resistance^59^. The signal obtained using this method has previously been studied for pain monitoring and has shown a significant correlation with acute pain intensity in healthy subjects^23^.

The second method, the endosomatic method, measures electrical skin potentials generated internally without applying an external current. This method is mainly used to measure *Skin Potential (SP)*^59^. This parameter, however, has shown no statistical correlation with acute pain in healthy volunteers^60^.

The EDA signal can be separated into its tonic (basal) and phasic components. The tonic component, called level (L), reflects the signal’s slow, almost DC variation (<0.05 Hz). The phasic component, named response (R), corresponds to the rapid variations of the signal (0.05-5 Hz). The *Skin Conductance Response (SCR)* is then the phasic component, and the *Skin Conductance Level (SCL)* is the tonic component of the EDA signal acquired using the exosomatic technique^59,61^. This last component has been attributed to a positive correlation with acute pain states^62^.

It is important to note that the decomposition techniques employed to extract the phasic and tonic components of the SC signal play a crucial role in the feasibility of those features as markers of pain. In this context, Posada-Quintero et al. (2021) discovered that features obtained through the sparse deconvolution approach (sparsEDA) exhibit greater classification power in distinguishing between pain and no pain states than those derived from the convex optimisation approach (cvxEDA)^61^. Furthermore, dynamic causal modelling (DCM) has been found to provide an SCR measure that is slightly more sensitive to acute pain levels when compared to sparsEDA, cvxEDA, and other methods like continuous decomposition (CDA) analysis and discrete decomposition analysis (DDA); it is also computationally demanding, making it less suitable for real-time applications^63^. In contrast to those findings, most articles reported the use of cvxEDA for SCR and/or SCL extraction^36,61,64–66^.

Most of the morphological features of the EDA signal are extracted from the SCR. One of them is the Amplitude of *the SCR (SCR_Amp)*, which exhibits a positive statistical correlation with acute pain intensity in healthy volunteers^60^. The time interval between the onset of a new electrical variation in the SCR signal and the point at which it reaches its peak is known as the SCR rise time (SCR_Trise). Significant differences in this parameter have been identified between different acute pain intensities in healthy subjects^60^.

The derivative of the phasic component (dPhEDA) has also been presented as a potential marker for pain assessment, showing a positive correlation with pain sensation and stimulus^61^. Conversely, SCR latency, sum, average, and integrated ISCR -defined as the area of the detected phasic activity- have shown no statistically significant correlation with pain in chronic cancer patients^32^.

Researchers have also investigated time-series features. For example, Pouromran et al. (2021) analysed 22 features related to value distribution and outliers, simple temporal statistics, linear autocorrelation, successive differences, and fluctuation scaling properties. They identified three key features to train models designed to assess pain: the time interval between successive extreme events above the mean and below the mean, and the exponential fit to successive distances in 2-dimensional embedding space^67^.

Moreover, Posada-Quintero et al. (2021)^68^ introduced a new index of sympathetic activity based on time-frequency spectral analysis of the EDA signal, named the Time-Varying EDA Index of Sympathetic Control (TVSymp). A modified version, MTVSymp, is defined as the difference between TVSymp at a time (t) and the mean value of TVSymp over the previous five seconds. Both versions of the index have displayed a positive correlation with pain sensation and stimulus in healthy volunteers^61^. In contrast, TVSymp has shown a moderate positive correlation with acute tooth pain during cold stimulation but a weak correlation when electric pulp testing (EPT) was performed^69^.

Furthermore, the time-series spectrum of the SCR signal (TFS-phEDA) and its power coefficients have been reported to improve the performance of deep-learning pain detection models in comparison to models only using the phasic component (SCR) of the signal^66^.

Finally, it has been suggested that different information about pain perception may be extracted from both endosomatic and exosomatic EDA. Bari et al. (2018) proposed that the Skin Potential Relative Early Turn (SPRET) could serve as a score to predict pain sensation. This score is calculated using measures from both endosomatic and exosomatic EDA^60^. Equation 4 shows the formula to calculate this parameter, where SPR refers to the Skin Potential Response obtained using the endosomatic signal.

4 $${SPRET}=\frac{{Time\; of\; SCR\; peak}-{Time\; of\; SPR\; peak}}{{Time\; from\; SCR\; onset\; to\; SCR\; peak}}* 100$$

Equation 4*Mathematical representation of the Skin Potential Relative Early Turn (SPRET)*.

### Electroencephalography (EEG)

Electroencephalography is a technique that measures electrical activity in the brain. This is done by using a set of electrodes attached to the scalp, which record the electrical currents generated by cortical neurons. The electrodes are placed on the scalp in specific locations according to the standardised international 10-20 system to ensure consistent results across different EEG subjects. Each electrode placement point is labelled with a letter indicating the underlying brain region: ‘F’ for Frontal, ‘T’ for Temporal, ‘P’ for Parietal, and ‘O’ for Occipital. The numbers associated with each label indicate the brain hemisphere, with odd numbers representing the left and even numbers representing the right hemisphere^70^.

Changes in neural activity within the brain lead to fluctuations in the EEG signal amplitude over time. A power spectral analysis is typically performed to monitor these changes across signal frequency ranges. This method decomposes the EEG time signals into their constituent frequencies^71^. The EEG signals contain five principal frequency bands, each related to a specific brain function: *Delta (~1–4 Hz), Theta (~4–8 Hz), Alpha (~8–13 Hz), Beta (~13-30 Hz), low Gamma (~30–50 Hz), and high Gamma (~52–100 Hz)*^23,72^.

In comparison to other physiological signals, EEG directly shows neuronal activation associated with pain sensation, providing an advanced method for the processing of complex pain information^71,73^. Lin et al. (2022) identified a significant increase in the EEG power density around the parietal area over all bands and a significant decrease around the central parietal area in alpha, beta and gamma bands associated with pain increase in healthy volunteers^23^. Okolo & Omurtag (2018) reported a statistically significant decrease throughout the cortex for all bands associated with increasing pain intensity in healthy volunteers^71^.

Zhou et al. (2020) studied how frontal function preservation influences pain tolerance in healthy individuals as they age. They found that ageing and cognitive performance impact theta and low gamma brain wave activity, suggesting that these characteristics can affect pain tolerance^72^. In contrast, Wang et al. (2020) found that there are minimal significant differences in the frontal and temporal regions in healthy subjects undergoing acute pain sensation^74^.

Furthermore, Tripanpitak et al. (2020) introduced a method for signal feature extraction using a non-linear analysis to obtain information from the complex dynamic characteristics of brain waves. They explored various Fractal Dimension (FD) computational techniques and identified that the feature combinations derived from the correlation dimension, using a moving variance approach, yielded the best results^75^.

Later, Feng et al. (2021) studied the alpha-band oscillation changes between closed and open eyes conditions as a potential pain biomarker, observing a linear relationship between the parameter and perceived chronic pain intensity^76^.

Some indicators derived from EEG signals have been proposed for pain assessment. For example, Naro et al. (2017) examined the potential of the *interpeak interval (IPI)* variability between the N2 and P2 components of *laser-evoked potentials (LEP)* as an acute pain biomarker in patients with disorders of consciousness. Their findings indicated a statistically significant negative correlation between this marker and self-reported pain levels as measured by the Nociception Coma Scale-Revised (NCS-R)^77^.

Similarly, An et al. (2017) introduced the *pain index (Pi)* for pain recognition. The index gives a value between 0 and 100, calculated by taking the frequency band of the brain waves using a wavelength algorithm. The study found a statistically positive correlation between the Pi and the self-assessment pain score in chronic pain patients^73^.

In contrast, the *Bispectral Index (BIS)*, commonly used in clinical settings to evaluate the level of consciousness in the cerebral cortex, has demonstrated no significant correlation with postoperative pain, indicating its unsuitability as a pain marker^51^.

### Blood Pressure (BP)

Researchers have found a statistically significant increase in blood pressure associated with acute pain increase in healthy volunteers undergoing electrocutaneous^49,50^ and cold^23^ stimulation. Yet, Ling et al.^41^ discussed the unreliability of using this marker in individuals taking antihypertensive agents such as β-blockers. These agents can suppress the increase of heart and blood pressure relative to pain stimuli. They found no correlation between those markers and the reported pain intensity in postoperative patients.

### Functional Near-Infrared Spectroscopy (fNIRS)

Researchers have shown interest in Functional Near-Infrared Spectroscopy to measure the variability of the nervous system associated with pain states. This optical technique measures changes in oxyhemoglobin (HbO) and deoxyhemoglobin concentrations^78^.

Pollonini et al. (2020) used this neuroimaging technique to explore the efficacy of non-pharmacological pain treatment for osteoarthritis patients that combines transcranial direct current stimulation (tDCS) and mindfulness-based meditation (MBM)^79^. Similarly, Du et al. (2023) employed this technique to study the hemodynamic effects of Transcutaneous Electrical Nerve Stimulation (TENS) for pain management in chronic pain patients, revealing that variation in pain sensation is associated with HbO levels in the prefrontal cortex^78^. Most recently, Khan et al. (2024) used the same TENS method, in this case, for pain stimulation in healthy volunteers, integrating ΔHbO2 and ΔHHB features^80^.

### Signal combination features

Matsubara et al. (2018) proposed a log-linearised peripheral arterial viscoelastic model, named the *Arterial Stiffness Index (β)*, that considers the nonlinear relationship between arterial wall impedance and blood pressure. The model uses the signals for PPG, ECG, and BP^49^. This model could accurately detect pain vs no-pain states^49^ showing a positive correlation with acute pain in healthy volunteers and with brain activity evoked by painful stimuli^50^.

*Pulse Transit Time (PTT)* refers to the time it takes for a pulse wave to travel from the heart to the peripheral arteries^45,81^. This parameter combines the ECG and PPG signals to calculate the pulse wave propagation time through the blood vessels. Research by Van Velzen et al. (2015) found that PTT decreased in response to painful stimuli and remains reduced even after those stimuli are reduced; while the marker’s immediate variation is slight, it becomes more significant over time^45^.

### Other physiological techniques

Respiration Rate (Resp) is a physiological signal obtained through a sensor placed around the chest or abdomen. The strap experiences deformation due to the expansion of the rib cage, leading to alterations in the recorded voltage. This measurement has been researched for pain assessment; however, no statistical correlation has been found across different pain states in healthy volunteers^23^. Despite this, Luebke et al. (2023) proposed that this measure could be better suited for chronic pain patients, as the fluctuations in respiration caused by acute pain stimulation may be more significant in those with chronic pain than in healthy controls^82^.

Pupillometry refers to the measurement of variations in pupil size, pupillary reflexes, and Pupillary Unrest in Ambient Light (PUAL). This measurement is conducted using a portable infrared pupilometer. Pupil diameter has shown significant differences between different acute pain states in healthy volunteer^23^. In contrast, research using the amplitude variations of pupil oscillations, referred to as PUAL, has shown that postoperative pain reduction in chronic pain patients does not affect this parameter^83^.

### Algorithms for pain assessment models

This section will discuss the latest advancements in the implementation of Machine Learning (ML) algorithms for pain recognition and assessment. Table 3 summarizes the findings related to algorithms implemented for pain detection and pain intensity evaluation. It also includes results from the best-performing models in each study, providing an illustrative comparison. From the 36 papers collected, 26 implemented ML for the assessment of acute pain, using traditional ML techniques (*n* = 14), deep learning approaches (*n* = 11), and a combination of both (*n* = 1). Additionally, seven studies addressed perioperative or postoperative pain, with the majority (71.4%, *n* = 5) relying primarily on photoplethysmography (PPG) signals. Only two studies explored the use of machine learning for chronic pain assessment, both utilising shallow learning methods. One study conducted a comparative analysis across multiple pain types, targeting both acute and chronic pain.

**Table 3:** Machine Learning algorithms used for pain assessment grouped by type of pain assessed and algorithm classification

Type of Pain	Algorithm implemented		Pain Assessment Approach	Physiological Markers	Outcomes	Author, Year
**Acute**	**SL / DL**	BiLSTM-XGB	(M) Pain intensity (4 levels)	**EDA**. SCL (mean, SD, range, AUC) and SCR (mean, SD, range, peaks max, peaks min, peaks sum, peaks num, duration mean, slope mean, AUC) from cvxEDA, whole signals extracted	**BL vs P3**. Precision 0.87, recall 0.84, F1 0.86	Pouromran et al., 2022^65^
	**Shallow Learning (SL)**	SVR	(M) Pain intensity (scale)	**EDA**. Time interval between successive extreme events above the mean and below the mean, and exponential fit to successive distances in 2-dimensional embedding space	MAE 0.93 ± 0.21. RMSE 1.16 ± 0.23	Pouromran et al., 2021^67^
			(M) Stimulation intensity (5 levels)	**fNIRS**. 10 statistical features from ΔHbO and ΔHHB each	Accuracy 66.55%, sensitivity 93.8%, specificity 96.14, F1 score 96.98%	Khan et al., 2024^80^
		SVM	(C) Pain detection	**EDA**. range, std, max, q3, sum, mean, median, q1, lqr, min	Accuracy 93.2% ± 8, precision 94.6% ± 6, sensitivity 96.8% ± 3 F1-score 95.5% ± 3.	Fernandez-Rojas et al., 2023^57^
			(C) Pain intensity (4 levels)	**EMG**. Corrugator amplitude peak-to-peak, corrugator Shannon entropy. **ECG**.HRV R-R slope	**BL vs P3**. Accuracy 77.05%	Walter et al., 2014^24^
			(C) Pain intensity (4 levels)	**EMG**. Zygomaticus (similarity correlation, SD of mean vector, amplitude RMS, linearity lag dependence, variability variance), corrugator and trapezius (amplitude peak, similarity correlation, similarity mutual information)	**BL vs P3**. Accuracy 90.94%, sensitivity 92.24%, specificity 89.65%	Gruss et al., 2015^25^
			(M) Pain intensity (4 levels).	Not reported	Accuracy 81.39%	Chu et al., 2017^85^
		RFc	(C) Pain intensity (5 levels)	**EDA**. SCR, SCL, dPhEDA, TVSymp (Max, min, range, SD, interquartile range, mean, local maxima and minima for all)	**BL vs P4:** **Volunteers**. Accuracy 91.70% **Patients**. Accuracy 89.58%	Khan et al., 2023^82^
			(C) Stimuli detection	**EMG**. Reflex withdrawal (Ipsilateral and Contralateral Limb). **ECG**. HR. **PPG**. SPO2. **EEG**. Noxious-evoked brain activity. **Facial expression**	Accuracy 0.81 (0.70–0.89), AUC: 0.9 (0.78–0.95).	Van der Vaart., 2019^86^
			(M) Pain intensity (5 levels).	**EDA**. Mean, max, RMS, IQR, mean maxima	**BL vs P1**. Accuracy 86%	Aqajari et al., 2021^64^
			(C) Stimulus intensity (5 levels)	**ECG, EDA, EMG**. 155 feat. including amplitude, variability, stationarity, entropy, linearity, similarity and freq.	**BL vs P4**. Accuracy 92%	Albahdal et al., 2024^87^
		Logistic regression	(C) Stimulus intensity (5 levels)	**EMG** (zygomaticus). FF, ZCD. **EDA**. SCR (slope)	**BL vs P4**. Accuracy 80%. Sensitivity 78%. Specificity 83%	Sen & Pal, 2021^125^
			(M) Pain intensity (4 levels)	**ECG**. HRV (RRI, LF, HF). **PPG**. PPGA, ANSS, BL	Accuracy 50%, sensitivity 60%, specificity 72%	Jhang et al., 2021^31^
		LightGBM	(C) Pain detection	**ECG**. Frequency spectrum analysis	Accuracy 0.9. Specificity 0.91. Sensitivity 0.9 Precision 0.87. F1-Score 0.88. AUC 0.96.	Chu et al., 2024^91^
		Bi-layered NNs	(C) Pain intensity (3 levels)	**PPG**. Time domain and PRV features	**BL vs P2**. 69% accuracy, 83.33% sensitivity and 75% specificity.	Khan et al., 2023^93^
	**DL**	Transformer-based Architecture	(C) Pain detection	**ECG+FaceExp**	Accuracy 82.74%	Gkikas et al., 2024^121^
		TCAtt-PainNet	(C) Pain detection & intensity (5 levels)	**EDA**. 11 statistical features. **ECG**. HR, HRV (SDNN, RMSSD)	MAE 0.87 ± 0.19. RMSE 1.07 ± 0.21. R2 0.39 ± 0.24. ICC 0.56 ± 0.22	Jiang et al., 2024^101^
	**Deep Learning (DL)**	Personalised Module (SensMeasure) + Base NN with DynAtt	(C) Pain intensity (5 levels)	**EDA**. Max, average, AUC and SD of SCR and SCL from cvxEDA. Phasic driver (average rise time, number of peaks, max peak amplitude). **ECG**. HR(mean, median), HRV (SDNN, RMSSD, LF). **Sensitivity**. SensMeasure	**P0 vs P4**. MAE 0.93 ± 0.18. RMSE 1.12 ± 0.20 R^2 0.34 ± 0.23 ICC 0.50 ± 0.22.	Jiang et al., 2024^36^
		Parallel TCN-SBU-LSTM	(C) Pain detection	**EDA**. SCR, TFS-phEDA	Accuracy 93.1%, F1-score 77.8%, AUC 0.967, sensitivity 82.3%, specificity 95%	Pinzon-Arenas et al., 2023^66^
		MLPNN	(M) Stimulus intensity (3 levels)	**EDA**. SCR, SCL, and dPhEDA obtained using sparsEDA. TVSymp and MTVSymp	Accuracy 69.7%, R2 0.357, M-RMSE 0.936, M-MAE 0.762	Posada-Quintero et al., 2021^61^
		LSTM-SW with DF	(M) Pain intensity (4 levels)	**EDA**. 1st & 2nd derivative, min, max, mean, SD.	ICC 0.33, MSE 0.08	Othman et al., 2023^96^
		Late fusion CNNs	(C) Pain intensity (4 levels)	**EDA, EMG** (trapezius), **ECG**	**BL vs P4**. Accuracy 84.40 ± 14.43 %	Thiam et al., 2019^98^
		Kernel ELM-based classifier	(M) Pain intensity (4 levels)	**EEG**. Alpha band	Accuracy 68.90 ± 3.12 %	Yu et al,. 2020^94^
		DFB-ConvNets	(M) Pain intensity (3 levels)	**EEG**. Alpha, Gamma, and Beta bands.	Accuracy 97.37%, precision 96.05%, specificity 98.03%, sensitivity 96.06%, F1-Score 96.05%	Yu et al., 2020^99^
		Deep-Attentive-Recurrent-CNN	(M) Pain intensity (4 levels)	**EEG**. Spatial spectral-temporal signal analysis	Accuracy 92.14 %, F1 score 92.11 %	May et al., 2021^117^
		CNN_LSTM	(C) Pain intensity (4 levels)	**ECG, EDA**. 2D waveform	**NP-PL4**. Accuracy 94.12%. MSE 0.0588	Subramaniam & Dass, 2021^100^
**Acute/Chronic**	**DL**	LSMT	(M) Pain intensity (3 levels)	**EDA**. SCR and SCL (median, SD, min, max 1st and 2nd derivative)	Accuracy 0.89 ± 0.05, F1-Score 0.85 ± 0.06	Badura et al., 2024^109^
**Chronic**	**SL**	RFc	(M) Pain intensity (4 levels)	**PPG**. PPGA number of changes, PRV (PR min, PR max, PR median, SDNNI, SDANN, RMSSD)	Accuracy 0.768 ± 0.012. Specificity 0.869% ± 0.007, sensitivity 0.737 ± 0.016, F1-score 0.768 ± 0.012.	Patterson et al., 2023^88^
		Modified K-Means	(C) Brain microstate analysis (5 levels)	**EEG**. GFP peaks	N/A	May et al., 2021
**Perioperative**	**DL**	MLPNN	(M) Pain intensity	**ECG**. ANI	MAE 2.848 ± 0.308	Jean et al., 2022^97^
**Postoperative**	**SL**	Logistic Regression	(C) Pain detection	**PPG**. NPI	Accuracy 75.3%, AUC: 0.8018	Cho et al., 2018^53^
			(C) Pain detection	**PPG**. RMSSD-ACVonset/ACAbl, AV-Asys/Atotal, SD-RS	Accuracy 0.752, AUC 0.825	Seok et al., 2019^84^
			(C) Pain detection	**PPG**. ACVsys/ACAdia	Accuracy 79.50%, sensitivity 74.02%, specificity 85.99%	Yang et al., 2018^47^
		Linear SVM + KDE + weighted Bayesian fusion	(C) Pain detection	**EDA**. TSD triangular matrix (mean, SD, entropy). SCL (SD). Video. 20 discrete Aus, three head pose indicators.	Accuracy 90.91%, sensitivity 100%, specificity 81.82%	Susam et al., 2022^92^
	**DL**	DBN (Selective bagging)	(C) Pain detection (4 levels)	**PPG**. Pulse height, rise time, fall time, average HR, PRV (HF, LF, VLF, LF/HF, AVNN, SDNN, RMSSD, NN20, pNN20, NN50, pNN50)	Accuracy 86.79 %, AUC 0.841 ± 0.039	Lim, 2019^56^
		CNN	(C) Pain detection	**PPG**. 2D spectrogram. SPI	Accuracy 71%, sensitivity 68.1%, specificity 73.8%, AUC 0.757	Choi et al., 2021^107^

Each entry highlights the top-performing machine learning model reported in each paper, the physiological markers used to train the model and the associated pain assessment approach. In “Pain Assessment Approach,” (M) denotes a multiclass or index development approach, while (C) refers to binary classification. In “Algorithms Reported,” SL is shallow learning and DL is deep learning. The pain intensity level goes from no pain or baseline (BL) to the highest-level group, for example, fourth level (P3).

### Shallow Learning Algorithms

Logistic regression is the most straightforward machine learning approach. It is a linear model used for binary classification, represented as a continuous function (See Eq. 5) that retrieves a value between 0 and 1. Seok et al. (2019) used the regression approach to build a model that uses PPG waveform features to assess postoperative pain^84^.

The algorithm can also be used for classification approaches by focusing the training objective on obtaining a binary outcome. Yang et al. (2018) implemented this algorithm as a classification model to train a model that takes PPG waveform features to differentiate between pain and no-pain states in patients undergoing surgery^47^. Similarly, Jhang et al. (2021) implemented the model to calculate the probability of pain in healthy volunteers using HRV and PPG features.

5 $$f\left(x\right)=\frac{1}{1+{e}^{-k(x-{x}_{0)}}}$$

Equation 5*Mathematical representation of the Logistic Classification Function*.^47^

Linear Discriminant Analysis (LDA) is a linear binary classifier that uses a linear discriminant function to classify new data points. A hyperplane is constructed by finding a linear combination of features to separate or categorise two classes of objects or events^85^. Research has shown the use of this algorithm to develop binary classification models that differentiate between pain and no-pain states. Some aimed to analyse the predictive power of individual parameters extracted from PPG signals^47^. Chu et al. (2017) implemented this algorithm using a one-vs-rest strategy to assess different levels of pain using PPG, ECG and EDA signals as input^85^.

Random Forest (RF) is an algorithm that uses a collection of decision tree classifiers to categorise the input data into multiple classes that correspond to the branches of the trees. Van der Vaart et al. (2019) found that RF models to detect noxious stimulation in newborns showed better results using a multimodal approach instead of single-signal models^86^. A study conducted using the BioVid pain dataset showed that models trained using RF had better accuracy when compared to SVM, LR, DT, NB, KNNs^87^. Patterson et al. (2023) implemented this algorithm to train a model capable of assessing four different pain intensity states in chronic pain patients with SCS systems^88^.

*Extreme Gradient Boosting Regression (XGBoost)* is an ensemble technique based on the sequential addition of new models, or trees, to correct errors made by previous learning trees. It uses the gradient descent algorithm to optimise the loss function^67,89^. Light Gradient Boosting Machine (LightGBM) is a tree-based ensemble designed to overcome the efficiency and scalability difficulties of the XGBoost algorithm by implementing Gradient-based One-Side Sampling and Exclusive Feature Bundling^90^. Chu et al. (2024) implemented this algorithm to train a model that takes ECG signals and classifies pain and no pain states in women during labour, demonstrating better discriminant power compared to the models obtained using XGBoost, SnapDecision-Tree and SnapLogisticRegression algorithms^91^.

According to the literature review’s findings, one of the most commonly used shallow learning algorithms is the Support Vector Machine (SVM). It is a supervised machine learning algorithm, especially useful in binary Classification and Regression (SVR) applications. Some authors use this model to compare its performance with their proposed ML approaches. Others have adopted it as the primary algorithm for the development of models that incorporate PPG^85^, EEG^71^, and EDA^57^ signals alone or using a multimodal approach^57,67,85^.

Chu et al. (2017) found that this algorithm outperforms models trained with Linear Discriminant Analysis (LDA) and K-Nearest Neighbours (KNN) algorithms using ECG, PPG and EDA signals for acute pain intensity classification across multiple subjects and days^85^. Susam et al. (2022) proposed an architecture that combines a Linear SVM model with a Kernel Density Estimation (KDE) that processes the model’s output and weighted Bayesian fusion of class conditional distributions for maximum likelihood classification using EDA and Video as input^92^. Lin et al. (2022)

The SVM classifier algorithm has also been used as a cascade-based classification tree to differentiate among various pain states (non-binary approaches), employing unimodal inputs (solely fNIRS signals)^80^ and multimodal inputs^23^. This approach has shown better results when compared with KNN and Disc algorithms^80^.

### Deep learning algorithms

Artificial neural networks (ANNs) are designed based on biological neural structures to learn and identify complex patterns within data. A typical feedforward neural network is structured with input, hidden, and output layers. The input layer receives the feature data and transmits it to the hidden layers, where the input weights are adjusted for optimisation. Ultimately, the processed information is sent to the output layer for final results^56,93^.

Extreme Learning Machine (ELM) is an algorithm designed for single-layer feedforward neural networks (SLFNs). This approach involves the random initialisation of the weights and biases of the hidden layer, followed by analytically determining the output weights through least-squares optimisation. Yu et al. (2020) studied the implementation of an ELM classifier and a kernel ELM classifier to classify four different acute pain states. Using EEG frequency bands. Their findings suggest that the kernel ELM classifier could be better for the task, even compared to the SVM model’s performance. Additionally, they identified that the Alpha and Beta bands were the most effective for pain prediction using these algorithms^94^.

Recurrent Neural Networks (RNNs) are designed to capture temporal relationships in sequential data. They are particularly beneficial for natural language processing and time series modelling. RNNs retain model outputs in internal memory and use them as additional input variables for predicting subsequent samples. *Long Short-Term Memory (LSTM)* algorithms are a specialised type of RNN that introduces a data-forgetting mechanism to overcome vanishing gradient problems, making it suitable for analysing temporal patterns over long sequences^65^.

Othman et al. (2022) analysed the performance of the LSTM algorithm, including a variation that incorporates sample weighting *(LSTM-SW)* across uni-, bi-, and multimodal approaches. Their findings suggest that the LSTM-SW algorithm is better for models combining two different modalities, namely EDA and Face Expression, in continuous pain intensity evaluation^95^. Furthermore, a combination of individually trained classification LSTM-SW models with *Decision Fusion (DF)* has also been implemented to increase performance in continuous pain monitoring^96^

Bidirectional LSTM (BiLSTM) algorithms have also been implemented independently and combined with XGBoost (BiLSTM-XGB) for a multiclass classification architecture using EDA signals, finding that BiLSTM-XGB using both whole signals and extracted features as input outperformed the model trained using BiLSTM alone^65^.

*Multilayer Perceptron Neural Networks (MLPNN)* is an algorithm that models biological brain structures. One input layer, at least one hidden layer, and an output layer according to a hierarchy. Weights indicate the degree of connection between each layer^56^. Jean et al. (2022) compared models derived from MLPNN and LSTM algorithms using the Analgesia Nociception Index as input. They found that the outcome score of the MLPNN model exhibited the strongest correlation with the Expert Assessment Patient Score (EAPS) used as a reference^97^.

In contrast, Lim et al. (2019) proposed the implementation of a Deep Belief Network (DBN) to model the complex relationship between physiological signals (PPG) and self-reported pain, demonstrating that this approach performs better than MLPNN. DBN is a probabilistic generative model built by stacking Restricted Boltzmann Machines (RBMs) layers. The process starts with pre-training using unsupervised learning to initialise the weights and bias of the model and supervised fine-tuning using the back-propagation algorithm of the MLPNN to minimise the error^56^.

*Convolutional Neural Networks (CNNs or ConvNets)* are algorithms inspired by biological NNs. They are designed to automatically and adaptively learn spatial hierarchies of features, also known as patterns, from visual data. CNNs’ architecture consists of layers processing the input data, with different layer types serving specific functions. The core layers, called Convolutional Layers, contain a set of neurons that scan the input sequentially and extract a particular pattern of information to build a set of feature maps^98^.

Several variations of this model have been implemented. Yu et al. (2020) successfully implemented diverse frequency band CNNs (DFB-ConvNets) models that extract features from each one of the bands that compose the EEG signal to classify acute pain between 3 different states^99^.

Subramaniam & Dass (2021) proposed a hybrid approach using CNN in combination with the LSTM algorithm named CNN_LSTM. This approach aimed to leverage the spatial feature extraction capabilities of the CNNs, along with the temporal sequence modelling ability of the LSTM to train models for binary classification between different pain states using ECG and EDA signals^100^. These algorithms have also been used in a parallel architecture incorporating a Temporal Convolutional Network (TCN) alongside a Stacked Bidirectional and Unidirectional LSTM (SBU-LSTM). This combined model, named Parallel TCN-SBU-LSTM, was used to detect acute pain using EDA signals^66^.

Moreover, Jiang et al. (2024) proposed an architecture that implemented a hybrid attention approach for pain intensity assessment using ECG and EDA signals. The algorithm contains a deep feature extraction module, temporal and channel attention modules, and a base module that uses temporal-channel attention weights to calculate the output^101^.

### Metaheuristic hybrid approach

*Genetic Algorithms* fall into this category. A stochastic algorithm based on an optimisation criterion that simulates biological evolution selects fitter sets of solutions (also called chromosomes or individuals) in a population at each generation. Feature selection has an exponential search space, making the genetic algorithm a potential fit for this data analysis stage. Chu et al. (2017) implemented this algorithm alongside the Principal Component Analysis (PCA) to perform feature selection and reduction among 12 statistical features, each from PPG, ECG and EDA signals^85^.

## Discussion

This literature review analysed the state of the art in pain assessment using physiological signals. While many physiological signals are currently explored for pain assessment, EDA has gathered considerable interest among other physiological signals for acute pain assessment in healthy subjects. A study comparing 112 features extracted from EDA, PPG and Resp signals found that statistical features from EDA are the most suitable for binary classification and pain intensity assessment in healthy subjects^57^. Similarly, studies conducted using the BioVid Heat Pain database have revealed that EDA is the most information-rich signal, compared with EMG and ECG signals, for pain classification and continuous pain intensity prediction in healthy volunteers^67,98^. Still, there is not enough evidence regarding the usefulness of this signal for chronic pain assessment paradigms.

Similarly, PPG signals show a high potential for use in pain intensity assessment. Nevertheless, there is still no conclusive evidence for the PPG features’ behaviour, as they show contrasting correlation feature behaviour, suggesting variability associated with different pain types. Furthermore, contrary to EDA, EMG, and ECG signals, before October 2024, there was no open-access pain database that included PPG signals for pain study, shortening the possibility of further analysing the potential of PPG for pain assessment.

Several physiological signals show strong potential for clinical translation in pain assessment; however, increased research focus is needed in this area, particularly for chronic pain. Of the studies reviewed, only 18 addressed chronic pain scenarios, with seven employing brain activity-related signals (EEG and fNIRS). Among these, EEG emerged as the most used modality, reflecting its value in capturing cortical responses to pain. While EEG offers important insights into the brain regions involved in pain processing, its use in long-term or continuous monitoring remains limited due to hardware complexity, susceptibility to artefacts, and poor wearability.

In contrast, HRV derived from ECG offers a more practical solution for wearable applications, reflecting autonomic responses to pain. However, HRV remains an indirect and non-specific marker, sensitive to confounding factors such as stress, respiration, physical activity, and emotional state. Its reliability in chronic pain contexts also requires further investigation.

Similarly, PPG signals—currently the most widely used in perioperative and postoperative monitoring—hold strong potential for broader application beyond surgical or ICU settings. PRV, extracted from PPG, presents a promising alternative to HRV, showing comparable trends under resting and controlled conditions, with the added advantage of easier integration into low-power wearable devices. However, like HRV, PRV is sensitive to multiple physiological and environmental confounders, and its correlation with HRV under dynamic or uncontrolled conditions has yet to be fully validated. This limits its current effectiveness in conscious and active states, highlighting the need for more targeted research before it can be reliably used in diverse clinical scenarios.

Likewise, the non-invasive nature and ease of integration into wearable devices of EDA sensors make it a practical choice for real-time monitoring in controlled settings, such as during experimental pain induction or perioperative care. However, the use of EDA in chronic pain scenarios remains limited, with relatively few studies exploring its reliability or clinical relevance in long-term conditions.

Overall, the combination of multiple modalities, such as ECG and PPG, or EDA and EMG, often yields improved accuracy compared to single-signal approaches, suggesting that multimodal strategies may be more promising for real-world applications. Despite these promising findings, several barriers hinder the translation of these approaches into routine clinical practice. These include high interindividual variability in physiological responses to pain and a lack of validation across diverse patient populations and clinical conditions, which limit generalizability. Further research is necessary to gain a comprehensive understanding of how different types of pain affect signal measurements, considering autonomic nervous system dysregulation^102^ and structural and functional brain alterations that can appear among different types of chronic pain^37^.

Generally, models and statistical analyses for various painful conditions rely on the self-assessment pain scale, i.e., the Numerical Rating Scale (NRS) or Visual Analogue Scale (VAS), as a measure of the actual pain reported by individuals. However, there is a subjective psychological component that affects the self-reported pain intensity, influenced by different social and psychological factors like anxiety, pain catastrophising and pain sensitivity^3,6,103^.

As a result, this approach could potentially introduce bias to the models when self-reported tools are used as the standard assessment methods for treatment and research purposes. Additionally, given that self-reporting scales and physiological signals may reflect different dimensions of pain, they are less likely to show a strong correlation when other measures that also reflect the subjective aspect are not incorporated into the analysis.

Moreover, psychophysical studies have demonstrated that there are gender differences in pain perception among adults, and it is necessary to carefully consider the interactions between gender and age in pain research^104^. Despite this evidence, only a limited number of studies have explored the role of gender, age and psychological phenomena like pain catastrophising and pain sensitivity in physiological signal variability and bias induction in pain assessment through self-reporting^3,26,36,40,79,100,103^. It is essential to address this issue during the model training process. One potential approach is to incorporate subject profiling as part of the data input, which could help mitigate the accuracy bias associated with interindividual variability.

Few measurements effectively predict pain intensity as a level indicator^67^. Most methodologies for pain assessment are based on binary classification, which categorises pain and no-pain states or between two different pain levels, like no-pain and low-pain or low-pain and high-pain. Unfortunately, this methodology often exhibits low performance in assessing mild and even moderate pain states. Additionally, this model is unsuitable for most clinical and research applications, where treatment relies on a fairly accurate objective analysis of pain. Thus, the main challenge remains in the objective quantification of pain intensity.

Given that pain is a multidimensional experience, multiple authors have explored combining different physiological signals and information sources, aiming to overcome the limitations of each signal. This approach aims to improve the sensitivity and specificity of the designed models for pain assessment. For instance, studies comparing unimodal and multimodal approaches generally show that multimodal methods enhance model outcomes, with some improvements being minimal while others are significant^57,67,98^. However, while taking this approach, it is essential to maintain a balance between incorporating more than one signal to enhance a model’s predictive capabilities and keeping the complexity of the technology manageable for practical, real-world applications.

In brief, this paper has reviewed the current state-of-the-art methodologies and techniques for the assessment of pain using physiological signals. Two main areas were addressed. A comprehensive analysis of the behaviour of different signal features related to pain states highlighted the differences among different pain types. It examined the current advances in machine learning approaches for the identification and measurement of pain using physiological signals. The review intended to highlight the currently available objective methods of acute and chronic pain assessment and the need to substantiate a method with better sensitivity and validity for clinical and research purposes.

It is important to note that no attempts were made to identify or translate non-English-language publications, which may have limited the inclusion of some relevant studies in this review. Also, publication bias may have occurred since only journal articles were included. Moreover, including only studies published since 2014 considerably restricted the number of studies analysed in this review. While this restriction reduced the total number of studies, it allowed us to focus on literature aligned with technological advancements from the past decade. Finally, despite our comprehensive search, we acknowledge that some relevant publications may not have been captured due to limitations in indexing and keywording. This is an inherent limitation of all literature reviews, including scoping reviews.

## Methods

### Review framework

The review was developed in accordance with the PRISMA extension for Scoping Reviews (PRISMA-ScR) guidelines^105^. The primary research question guiding this review was: *“What are the current state-of-the-art methods for* ***pain assessment*** *using* ***physiological signals****?*” In line with this question, the review aimed to analyse the behaviour of physiological signals across different pain types, highlighting key differences in signal patterns and features reported in the literature, and to summarise the machine learning models implemented in studies using physiological signals for pain assessment, including the modelling approaches, features used, and reported outcomes.

### Literature search strategy

**Fig. 4:**
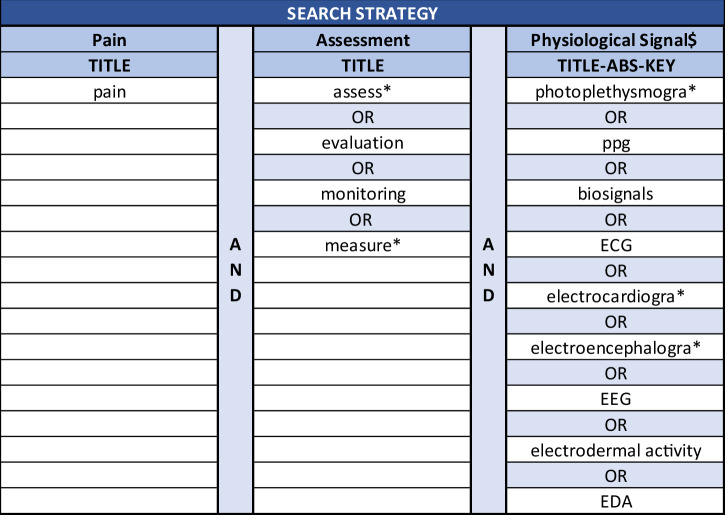
Search strategy implemented. The figure illustrates the search strategy used for the systematic review. Three categories of terms were combined: pain, assessment, and physiological signals. Terms for pain and assessment were restricted to article titles, while physiological signals were searched in titles, abstracts, and keywords. The dollar sign in “Signal$” indicates truncation, allowing the inclusion of variations such as “signal” and “signals”.

A literature search was conducted using four online databases: PubMed, Embase, Scopus, and Web of Science. Since this review focuses on analysing the latest technological advancements in pain assessment using these signals, only studies published within the last ten years (2014-2024) were included. The search was done in April 2024 and updated in October 2024. The strategy implemented was structured as shown in Fig. 4 where the keywords respond to the research question and the previously introduced aims.

### Eligibility criteria

The criteria for inclusion were as follows: (1) Studies that either (a) report statistically significant relationships or correlations between physiological signal variations and pain intensity or pain states, or (b) describe the development of machine learning models using physiological signals to assess pain. (3) Articles with full text accessible, (4) articles at the final stage of publication, (5) papers with available abstracts. All types of pain were included to create a comparative review that examines differences in signal patterns, feature behaviour, and trends in pain assessment methods across various pain types.

The criteria for exclusion were as follows: (1) conference abstracts, conference papers, letters, editorials, case-control studies, summaries, expert opinions, and comments; (2) studies conducted in animals; (3) studies not using physiological signals; (4) articles not available in English; and (5) full text not available/retrievable. Conference papers were excluded from this review because they often lack detailed methodology and complete experimental data. To ensure the inclusion of robust, fully developed studies that provide a complete picture of the research conducted, only peer-reviewed journal articles and full-length studies were considered.

### Data charting and study selection

All search results were imported into Microsoft Excel for deduplication and screening. Reasons for exclusion at the full-text stage were recorded in an Excel spreadsheet. No specialised systematic review software was used. First, titles and abstracts were reviewed to exclude studies that were unrelated based on the predefined eligibility criteria. Second, the full texts of all potentially relevant articles were retrieved and assessed for final inclusion. No significant disagreements arose during the screening process. Minor uncertainties regarding eligibility were discussed and resolved by consensus among all authors.

### Data extraction and synthesis

From each included study, the following information was extracted where reported: type of pain assessed, study population (volunteers or patients), sample size, participants’ age and sex distribution, methodological approach (pain modulation method), self-report instruments used as the reference for pain, whether the data comes from a publicly available database, physiological signals recorded, extracted physiological markers, and reported behaviour of signals or features.

For studies implementing machine learning models, we additionally extracted: the type of machine learning model applied, the modelling approach (e.g., pain detection, pain level classification including the number of levels, or pain quantification), features or markers used for training, testing and validation methodology, and reported model outcomes.

Finally, the extracted data were synthesised descriptively and grouped into two main thematic domains: 1) physiological signals used for pain assessment or modulation, and 2) machine learning models implemented for analysis or prediction. Findings are presented in summary tables and narrative form. Given the scoping nature of the review, no meta-analysis or formal critical appraisal was conducted; however, basic indicators of study context are reported to provide context for interpreting the evidence.

It is important to note that in this review, a feature or effect is described as statistically significant only if the original study explicitly reported a p-value less than or equal to 0.05. This ensures that statements about significance directly reflect the findings reported by the authors of the included studies.

## Supplementary information

PRISMA-ScR-Fillable-Checklist.

## Data Availability

The datasets generated and/or analysed during the current study are either published in this article or available on the Zenodo research repository, 10.5281/zenodo.17135805.
